# Special Issue “Role of Glia in Human Health and Disease”

**DOI:** 10.3390/ijms27209151

**Published:** 2026-10-15

**Authors:** Daniele Lana, Hajime Hirase

**Affiliations:** 1 Department of Health Sciences, University of Florence, 50139 Florence, Italy; 2 Center for Translational Neuromedicine, Faculty of Medical and Health Sciences, University of Copenhagen, 2200 Copenhagen, Denmark; 3 Center for Translational Neuromedicine, University of Rochester Medical Center, Rochester, NY 14642, USA

## 1. Introduction 

This Special Issue, “Role of Glia in Human Health and Disease”, is a collection of eleven contributions: eight original research articles and three reviews. The original studies employ diverse experimental approaches, including electrophysiology, multiphoton imaging, mass spectrometry-based proteomics, chemogenetics, and behavioral analysis. Together, these contributions highlight the many ways in which glial cells regulate neuronal function and examine how glial dysfunction may contribute to the initiation or progression of CNS disorders.

## 2. Alzheimer’s Disease and Neurodegeneration

Alzheimer’s disease (AD) research has traditionally focused heavily on the cerebral cortex and hippocampus [1]. However, motor deficits frequently manifest as comorbidities during disease progression [2], necessitating an evaluation of spinal cord pathology. Wang et al. (2026) (contribution 1) sought to investigate spinal cord alterations in the 5xFAD transgenic mouse model. The authors quantified beta-amyloid deposition, neuroinflammatory marker expression, and cholesterol metabolism across the cervical, thoracic, and lumbar segments of mice sacrificed at three and nine months of age. While neuronal loss was not detectable, an age-dependent accumulation of amyloid plaques was found in the gray matter, particularly within the thoracic and lumbar segments. Notably, reactive astrocytes and microglia colocalized within these affected regions. The RT-PCR analysis of lipid homeostasis-related genes indicated a marked downregulation of *Abca1* and *Cyp46a1*, which was supported by significant free cholesterol accumulation in the spinal white matter. Interestingly, astrocytic reactivity was more pronounced in female mice, whereas specific regional cholesterol accumulation was higher in males. These results support the notion that the spinal cord is a highly susceptible region in AD.

Microglia are increasingly recognized as key mediators of neuroinflammation and neuronal dysfunction in AD [3]. Soto-Mercado et al. (2025) (contribution 2) developed a humanized in vitro model of microglia–neuron interactions by generating induced microglia-like cells (iMGs) from peripheral blood monocytes obtained from patients with familial or sporadic Alzheimer’s disease (AD). These iMGs were exposed to conditioned medium from, or co-cultured with, cholinergic-like neurons carrying the PSEN1 E280A mutation, enabling the direct assessment of microglial responses to neurons with a defined AD-associated mutation. Patient-derived iMGs displayed marked functional abnormalities, including impaired phagocytosis, while co-culture with PSEN1 E280A neurons promoted an M1-like pro-inflammatory phenotype and increased the secretion of pro-inflammatory cytokines. In turn, the iMGs aggravated neuronal pathology, including abnormal tau phosphorylation and apoptosis. This study provides evidence for a self-amplifying, reciprocal interaction between dysfunctional microglia and PSEN1 E280A cholinergic-like neurons, thereby promoting sustained neuroinflammation and neuronal damage. 

## 3. Neurodevelopmental and Circadian Disorders

Astrocytic heterogeneity presents a significant barrier in translating rodent models to human neurobiology, particularly concerning interlaminar astrocytes (ILAs), a primate-specific subtype residing in cortical layer I with long processes extending into deeper layers [4]. Anding et al. (2025) (contribution 3) bypassed this structural limitation by engineering a chimeric mouse model engrafted with human-induced pluripotent stem cell (hiPSC)-derived astrocytes. This system allowed for the examination of human ILAs within an intact mammalian neural circuit. The researchers focused on Fragile X Syndrome (FXS), a prevalent genetic cause of autism spectrum disorder. Utilizing multiphoton microscopy and genetically encoded calcium indicators, the study mapped the calcium signaling dynamics of normal and FXS-derived ILAs in response to canonical neurotransmitters and found significant hyperexcitability characterized by aberrant, high-frequency cytosolic calcium oscillations. Moreover, the authors documented increases in the turnover rate of dendritic spines in the vicinity of the FXS ILAs. These findings implicate primate-specific interlaminar astrocytes in the dysregulation of synaptic plasticity, extending the established view of astrocytes as modulators of synaptic plasticity and learning through activity-dependent signaling mechanisms [5,6,7,8].

Beyond their homeostatic roles, astrocytes actively modulate neural circuits involved in movement, neuromodulators, and behavioral state [9,10]. The influence of astrocytic activity on cytoarchitectural organization and motor control was structurally assessed by Maamrah et al. (2025) (contribution 4). The researchers utilized Designer Receptors Exclusively Activated by Designer Drugs (DREADD) technology [11] to induce chronic chemogenetic activation of the Gq G-protein-coupled receptor signaling in mesopontine astrocytes in mice. The authors found that chronic astrocytic activation resulted in substantial motor and circadian abnormalities. The test subjects exhibited a significant reduction in the amplitude of the acoustic startle reflex and an abnormal increase in locomotor speed. Further detailed gait analysis confirmed mechanical alterations in movement, whereas spatial memory remained unaffected. Histological evaluations indicated that the sustained glial activation resulted in a decline in the local neuron density, establishing a causal relationship between localized glial overactivation, progressive palsy, and motor symptoms.

## 4. Epilepsy and Astrocytic Physiology

Astrocytes function as central regulators of excitatory neurotransmission controlling the release and reuptake of glutamate at the synapse [12,13]. Sarmadi et al. (2026) (contribution 5) investigated the specific contribution of volume-regulated anion channels (VRACs), whose pore is defined by the Leucine-Rich Repeat Containing 8a (LRRC8a) subunit, to the initiation of temporal lobe epilepsy. The authors leveraged an astrocyte-specific inducible LRRC8a knockout mouse model to demonstrate that a large portion of tonic NMDA receptor currents in CA1 pyramidal neurons originated from astrocytic VRAC. To evaluate pathological states, the mice were subjected to an intracortical kainate injection to induce status epilepticus. Slices analyzed four hours post-injection revealed little alteration in *Lrrc8a* mRNA levels. Furthermore, the DCPIB-sensitive tonic NMDA currents appeared to be unaltered. These electrophysiological measurements indicate that while LRRC8a channels govern physiological extracellular glutamate homeostasis, they do not upregulate their activity during the acute phase of experimental epilepsy.

The structural and functional states of astrocytes adjust dynamically to systemic physiological requirements [14]. Su et al. (2026) (contribution 6) provided a comprehensive molecular map of these adaptations by a quantitative proteomic and phosphoproteomic analysis of rat cortical astrocytes across three distinct brain states: active wakefulness, sleep, and sevoflurane anesthesia. The transition from wakefulness to both sleep and anesthesia induced a structural remodeling signature, represented by the downregulation of cytoskeletal intermediate filaments, including keratin types 1 and 10. Further, sevoflurane anesthesia was found to be biochemically distinct from sleep. The anesthetic state selectively suppressed beta-amyloid binding pathways and bicarbonate transport. At the post-translational level, sevoflurane uniquely downregulated the phosphorylation of Ser188 on the nuclear casein kinase and cyclin-dependent kinase substrate 1 (NUCKS1), an event linked to the cessation of specific cell cycle activities. By contrast, sleep increased the phosphorylation of the RNA splicing machinery. This work differentiates anesthesia from physiological sleep at the glial proteomics level.

## 5. Stress Responses and Environmental Toxicology

Environmental stressors precipitate severe psychiatric conditions [15]. Prevot et al. (2026) (contribution 7) investigated the temporal trajectory of behavioral deficits and cellular remodeling in the prefrontal cortex (PFC) of mice subjected to chronic restraint stress (CRS) for a period of 35 days. The authors established a chronological hierarchy of phenotypic manifestations through continuous behavioral tracking. Anxiety-like avoidance behaviors assessed by a PhenoTyper^®^ test emerged rapidly by day 7. By contrast, anhedonia-like behaviors, measured via sucrose preference, required the full 35 days. Next, the authors quantified molecular markers across different cellular compartments in the PFC, revealing a sequential degradation sequence. Synaptic integrity was compromised first, evidenced by an immediate reduction in vesicular glutamate transporter-1 (Vglut1) at day 7. The GABAergic inhibitory network deteriorated subsequently, with significant reductions in GAD67 occurring between days 14 and 21. The astrocytic compartment exhibited the most delayed response, with downregulation of glial fibrillary acidic protein (GFAP) registering at day 28. This compartmental temporal mapping demonstrates that stress-induced brain pathology is a progressive functional degradation where synaptic deterioration precedes structural astrocytic suppression.

The industrial application of engineered nanomaterials necessitates rigorous toxicological evaluation within the CNS [16]. Geiszelhardt et al. (2025) (contribution 8) investigated the specific neurotoxic mechanisms of nanosized titanium dioxide (TiO_2_), comparing the distinct mineralogical crystal structures of rutile and anatase in primary glial and neuronal–glial mixed cultures. The toxicological data demonstrated a high degree of structural and cell-type specificity. The rutile crystalline form induced severe, dose-dependent cytotoxicity and cell death in primary cortical astrocytes at concentrations exceeding 10 µg/mL within 24 hours. Microglial cells exhibited similar vulnerability to the rutile form. Conversely, hippocampus-derived glia demonstrated a higher resistance. The anatase form of TiO_2_ produced lower baseline toxicity across all evaluated cell types. Mechanistic assays indicated that rutile nanoparticle toxicity is primarily driven by the rapid induction of oxidative stress, which overwhelms the astrocytic antioxidant defense systems and induces structural membrane degradation.

## 6. Review Articles

Complementing the original research, this Special Issue includes three review articles that address distinct areas of translational glial biology: neonatal hypoxic–ischemic brain injury and neuroprotection [17], intercellular glial signaling in CNS homeostasis and disease [18], and microglial dysfunction in neurodegenerative and neurocognitive disorders [19]. These are well-established fields in which glial cells have emerged as important contributors to both tissue homeostasis and pathological responses. At the same time, rapid advances in our understanding of glial heterogeneity, cell–cell communication, and disease-associated cellular states, together with continued efforts to translate these mechanisms into therapeutic strategies, have substantially reshaped these areas in recent years. The three review articles are therefore particularly timely, providing updated perspectives on rapidly evolving aspects of translational glial biology.

Gebala et al. (2025) (contribution 9) systematically evaluate the current pharmacological landscape for treating the neurodevelopmental consequences of perinatal hypoxic–ischemic brain injury. The review details how the disruption of oxygen and blood flow precipitates massive excitotoxicity, oxidative stress, and neuroinflammation. The authors critically assess the molecular mechanisms of novel neuroprotective agents designed to augment standard therapeutic hypothermia, emphasizing drugs that target glial-mediated neuroinflammation and the prevention of delayed apoptotic cascades in the neonatal brain.

Bencsik et al. (2025) (contribution 10) show the potentiality of liquid–liquid phase separation (LLPS) in cell biology as a technique that can help to understand how cells organize biochemical reactions and structures through dynamic, membraneless organelles (MLOs) and gives a framework to interpret altered synaptic functions in various neurodevelopmental disorders. Authors explores the role of stress-induced MLOs in the nervous system, the molecular principles governing their formation, and how their dysfunction bridges the gap between environmental stress responses and neurodevelopmental impairment.

Zheng et al. (2025) (contribution 11) discuss the integration of microglial dysfunction within the broader frameworks of progressive neurocognitive disorders, including AD and associated virus-induced cognitive declines. The review summarizes evidence regarding the transition of microglia from physiological immune surveillance to pathological, hyper-reactive states driven by chronic antigen exposure and misfolded protein aggregates. The authors detail how sustained microglial activation disrupts localized synaptic pruning mechanisms, ultimately accelerating structural and cognitive degradation.

## 7. Conclusions

The original research articles and review articles in this Special Issue highlight the important contribution of precisely regulated glial function to central nervous system physiology. The findings presented elucidate the potential contribution of astrocytic and microglial dysfunction to brain-disease-related processes. The documentation of state-dependent molecular remodeling, temporal stress degradation, and specific toxicological vulnerabilities offers a robust empirical foundation for future neurobiological research. By identifying the exact molecular and temporal inflection points at which physiological glial support transitions into pathological neuroinflammation or metabolic failure, this article collection illustrates new possible therapeutic targets for the management of severe neurological, neurodevelopmental, and neurodegenerative disorders.
